# Serological Analysis and Drug Resistance of* Chlamydia pneumoniae* and* Mycoplasma pneumoniae* in 4500 Healthy Subjects in Shenzhen, China

**DOI:** 10.1155/2017/3120138

**Published:** 2017-09-19

**Authors:** Dong Du, Shuping Liao, Yan Wu, Yang Jiao, Di Wu, Weiqing Wu, Dapeng Yu

**Affiliations:** ^1^Shenzhen People's Hospital, Shenzhen, Guangdong, China; ^2^Shenzhen YHLO Biotech Co., Ltd., Shenzhen, Guangdong, China

## Abstract

**Objective:**

To understand the prevalence and distribution of* Chlamydia pneumoniae* (CP) and* Mycoplasma pneumoniae* (MP) in the population and to provide a basis for the prevention and treatment of respiratory tract infection.

**Methods:**

This study included a total of 4500 healthy subjects who were given physical examination in Shenzhen People's Hospital from January to December in 2016. Venous blood was drawn from people to detect the MP- and CP-specific IgG and IgM in the serum using chemiluminescence immunoassay (CLIA). The relationship of MP and CP infections with patient age, seasons, and percentage of infections was analyzed.

**Conclusion:**

CP and MP cause high rate of asymptomatic infection, which may be associated with the high incidence of CP and MP infection, especially in children and the elderly population. Therefore, the implementation of effective and practical prevention measures has become an urgent need. MP culture and drug sensitivity test should be performed as early as possible in patients with manifested MP infections in order to ensure timely and proper treatment and to reduce the emergence of drug-resistant strains.

## 1. Introduction

*Chlamydia pneumoniae* (CP) and* Mycoplasma pneumoniae* (MP) are essential atypical pathogens in community-acquired pneumonia (CAP) [1]. CP and MP have similar epidemiological and clinical characteristics, and the severity of pneumonia caused by the two pathogens varies depending on the age of patients [2]. Recent studies have shown that MP and CP may induce chronic cough (CC), one of the recurrent and refractory respiratory diseases, resulting in delayed cure. Although this disease normally does not incur serious consequences in the body, it can cause a variety of organ complications, affecting the patient's physical and mental health [3]. In addition, there also normally exist mixed infections with other bacteria [4]. In this study, the epidemic distribution of infection rates of CP and MP was investigated through detection of the serum antibodies of CP and MP in 4500 healthy people.* Mycoplasma* culture and drug resistance analysis were performed in* Mycoplasma*-positive patients.

## 2. Materials and Methods

### 2.1. General Information

The objects of this study were selected and divided into groups according to seasons and patient ages, from the healthy population given physical examination in the Medical Examination Department of Shenzhen People's Hospital from January to December in 2016: Group A, 1500 cases of children, aged between 4 and 14 with the average of 8.24 ± 2.86 years; Group B, 1500 cases of juniors, aged between 18 and 40 with the average of 28.24 ± 4.27 years; Group C, 1500 cases of seniors, aged between 60 and 80 with the average of 70.5 ± 3.5 years. Patients with clear immune system diseases, chronic pulmonary inflammatory diseases, and severe systemic diseases were excluded.

There are 1125 cases occurring in spring (Feb.–Apr.), 1125 cases in summer (May–Sept.), 1125 cases in autumn (Oct.-Nov.), and 1125 cases in winter (Dec.-Jan.).

It can be conclusively showed in Table 1.

### 2.2. Examination Methods

Venous blood (2 mL) was collected from each object after overnight fasting and mixed with anticoagulant. Blood samples were centrifuged at 3000*g* 4°C for 5 min, and the liquid supernatant was kept in a cryopreservation tube at −20°C for testing. IgM and IgG antibodies of MP and CP were detected by chemiluminescence immunoassay (CLIA) using commercial kits (Shenzhen YHLO Biotech Co., Ltd., Shenzhen, China) in accordance with the manufacturer's instructions.

Concrete procedure of the detection is listed as follows, which is a two-step indirect immunoassay using direct chemiluminometric technology.

1st incubation:* Mycoplasma pneumoniae* IgM in the sample and* Mycoplasma pneumoniae* antigen-coated paramagnetic particles react to form an antigen-antibody complex.

Wash: the unbound materials are washed away from the solid phase in a magnetic field.

2nd incubation: acridinium-labeled anti-human IgM conjugate is added to form an antigen-antibody-second-antibody complex.

Wash: the washing step is repeated.

Trigger of signal: Pretrigger and Trigger Solutions are added to the reaction mixture. The result of chemiluminescent reaction is measured as relative light units (RLUs). A direct relationship exists between the amount of* Mycoplasma pneumonia* IgM in the sample and the RLUs detected by the optical system of analyzer.

A comparison between RLU generated by the sample and the cut-off calculated by* Mycoplasma pneumonia* calibrator will be made automatically by the analyzer.

For MP IgM (+) population, throat swab specimens were collected and cultured immediately to determine the drug sensitivity using* Mycoplasma* rapid culture and drug sensitivity kit (Shenzhen YHLO Biotech., China). The MP microbes used the rapid growth factors in the culture medium to proliferate and decompose carbohydrates and subsequently to produce hydrogen ions, which reduced the pH value of the medium. As a result, the culture medium turned from red to yellow, and the liquid was clear, indicating the growth of MP. Based on the inhibitory effect on the microbes* in vitro* of the 6 antimicrobial agents (including erythromycin, roxithromycin, azithromycin, acetyl spiramycin, clindamycin, and clarithromycin, at high and low concentrations) in the drug sensitivity plate, the microbial drug resistance was analyzed. The drug sensitivity results were read as follows: two yellow wells indicate the sensitive; red low concentration well and yellow high concentration well indicate the intermediary; two red wells indicate the resistant.

### 2.3. Infection Diagnosis Criteria

Acute infection was IgM (+); chronic infection was IgM (+) and IgG (+); prior infection was IgM (−) and IgG (+).

### 2.4. Drug Sensitivity Determination Criteria

The rapid growth factor of the culture medium is used for microbes to proliferate and decompose the carbohydrates, and the hydrogen ions are produced to lower the pH value of the medium. Then the indicator in the medium is converted from the original red to the pale yellow. When the medium turns from red to yellow, and the liquid is clear, MP growth is demonstrated. The microbial drug resistance was analyzed utilizing the inhibitory effect on the microbes* in vitro* of the 6 antimicrobial agents in the drug sensitivity plate, including erythromycin, roxithromycin, azithromycin, acetyl spiramycin, clindamycin, and clarithromycin, at high and low concentrations. The drug sensitivity results were read as follows: two wells were yellow, indicating the sensitive; low concentration well was red, and high concentration well was yellow, indicating the intermediary; two wells were red, indicating the resistant.

### 2.5. Statistical Analysis

SPSS 13.0 was used for statistical analysis. Measurement data were presented as mean ± standard deviation. Two-tailed random sample *t*-test was used for numerical variables. Enumeration data were presented as percentage, and chi-square test was used. The difference was statistically significant with *P* < 0.05.

## 3. Results

### 3.1. Detection of MP and CP Infection in Each Group

Among the serum of 4500 healthy objects examined for CP by CLIA, IgM antibody was positive in 167 cases, IgG antibody was positive in 2232 cases, and IgM + IgG was positive in 65 cases. The infection rate of positive IgM + IgG of Group C was the highest among all three groups of A, B, and C. On the other hand, for MP, IgM antibody was positive in 102 cases, IgG antibody was positive in 1905 cases, and IgM + IgG was positive in 35 cases. For MP the infection rate of positive IgM + IgG of Group A was the highest among all three groups.

The infection rates, including IgM antibody positive rate, IgG antibody positive rate, and IgM + IgG positive rate, for MP and CP were different between groups with statistical significance (*P* < 0.05) (see Table 2).

### 3.2. Infections in Different Seasons

MP and CP infections were observed throughout the whole year, and the infection rates were different between different seasons, especially in winter which is the peak season (see Table 3).

### 3.3. MP Drug Sensitivity

The MP sensitivity to the 6 commonly used antimicrobial agents of erythromycin, roxithromycin, azithromycin, acetyl spiramycin, clindamycin, and clarithromycin was 42.15% (43/102), 54.90% (56/102), 66.67% (68/102), 68.63% (70/102), 67.64% (69/102), and 59.80% (61/102), respectively.

## 4. Discussion

CP and MP are atypical pathogenic microorganisms between bacteria and viruses. CP and MP have similar epidemiological and clinical characteristics, and the severity of pneumonia induced by CP and MP varies depending on the age. Recent studies have shown that MP and CP, mainly transmitted through respiratory droplets and causing infections of respiratory tract and lung, may induce chronic cough (CC), a recurrent and refractory respiratory system disease, which results in protracted cure. Although this disease normally does not result in serious consequences in the organism, it can cause a variety of organ complications, affecting the patient's physical and mental health [3]. In addition, there also normally exist mixed infections with other bacteria [4].

With a widespread of MP pneumonia in the world, the exact incidence rate varies due to different ages, regions, years, and prevalence. As reported by studies in China, the incidence rate is 10% to 20% in nonpopular years and 30% in popular years [5]. Different from viral or bacterial pneumonia, MP pneumonia is more easily to occur in winter. The early childhood infection rate is lower than the elderly, for the probable reason that the infection rate is indeed low or that the atypical infection symptoms at this age lead to missed diagnosis or misdiagnosis.

However, some scholars believe that the low infection rate in early childhood resulted from the relatively low immune response of infants. Through the analysis of MP serum in 4500 cases of healthy objects given physical examination, it is found that the infection rate in the group of children aged between 4 and 14 is the highest, up to 4.25%, which is in accordance with the above-mentioned conclusion that some infection symptoms at this age are atypical or some infections are asymptomatic. MP mainly infects respiratory tract, the epidemic outbreak, and prevalence of which occurs mainly in schools and other crowded places with lots of children. Infectious MP pneumonia seriously affects children's health with many severe extrapulmonary complications [6], so that controlling the occurrence and prevalence of this disease has become an urgent need for solving the problem. However, the pathogenesis of MP infection has not been clear yet so far for MP is widely distributed in natural world. Most experts believe that, in addition to directly causing host cell damage in the body through metabolites and toxins, MP infection also induces cellular immune function inhibition, humoral immune disorder, and autoimmune hyperfunction, resulting in body damage [5, 7].

MP has natural resistance to *β*-lactam antibiotics due to the lack of cell wall [8], but it is sensitive to antimicrobial agents that inhibit microbial protein synthesis, such as macrolides and tetracyclines, and that inhibit DNA replication such as quinolones. Tetracycline antibiotics have inhibitory effect on the development of children's bones and teeth [9], which are only suitable for elder children (over 8 years old). Quinolone antibiotics have some damage to the musculoskeletal system, which are used only when other antimicrobial agents are ineffective [7]. As a result, macrolide antibiotics have become the first choice for the treatment of MP in young children. However, macrolide-resistance MP (MRMP) has become increasing prevalent due to the overuse of antibiotics since it was first reported in Japan in 2001.

In 2001, Japanese scholar Okazaki [10] firstly extracted MP bacterial strain which has drug resistance to macrolide antibiotics from the lower respiratory specimens of children patients with pneumonia and bronchitis. Subsequently, the survey of Matsuoka [11] also showed that there are 13 bacterial strains drug-resistant to erythromycin in 76 MPs extracted from Japanese children patients, among which MIC (Minimum Inhibition Concentration) of erythromycin to 12 MPs is more than 256 g/ml. It has been confirmed that drug resistance to macrolide antibiotics of MP is mainly related to the mutation of Gene 23SrRNA in Area V, most commonly in the mutation of A2063G and followed by A2064G and A2617G. Rarely, mutation of ribosomal protein L4 or L22 may induce drug resistance to macrolide antibiotics [10–12]. Since 2007, it has been reported that clinical MP bacterial strain drug-resistant to macrolide antibiotics was found [10–12]. From the result of dynamic monitoring in Japan and France, total rate of MP drug resistance to macrolide antibiotics has been rising year by year, up to 30.6% in Japan in 2006 and 9.8% in France from 2005 to 2007.

Monitoring of MP drug resistance in China started late with first monitoring data publicly reported in 2009 from a few hospitals in Beijing and Shanghai. However, data show that the situation of MP drug resistance in China is more severe, percentage of which is up to 83% in Shanghai Huashan Hospital, 90% in Shanghai Children Hospital, and 92% in Beijing Friendship Hospital. Moreover, most of drug-resistant strains are in high level with the MIC value over 128 *μ*g/mL [10–12]. Great differences of clinical application in antibacterials exist in China for its vast territory and therefore the situation of MP drug resistance in Beijing and Shanghai cannot totally reflect the whole in China.

Collecting throat swab specimens and immediately using* Mycoplasma* rapid culture and drug sensitivity kit, this study analyzed the MP sensitivity to the 6 commonly used antimicrobial agents of erythromycin, roxithromycin, azithromycin, acetyl spiramycin, clindamycin, and clarithromycin, whose results were 42.15% (43/102), 54.90% (56/102), 66.67% (68/102), 68.63% (70/102), 67.64% (69/102), and 59.80% (61/102), respectively.

Abuse of antibiotic in recent years leads to the generation of drug-resistant strain to MP, causing difficulty in clinical treatment. Aiming at amides clindamycin, MP sensitivity of the statistical result in this study is 67.64% with fewer side-effect than erythromycin.

A great difference exists in sensitivity of 7 kinds of macrolides antibiotics in this study (*P* < 0.01), which may be related to the long-term clinical use of this mendicant in the specific area. However, there are some differences between* in vitro* susceptibility test and in vivo susceptibility, which is affected by route of medication, drug transport, or half-life period. The half-life period of azithromycin is up to 68 hours with clear target-cell effect, which may make use of phagocytic cells as carriers for drug movement to the inflamed part, to improve its concentration over sixfold. Therefore, the actual clinical curative effect of azithromycin may be more effective than the result* in vitro*. Additionally, azithromycin has been a choice drug of MP infection clinically. Other reports say the combined treatment of clindamycin and azithromycin is better than the individual use of azithromycin [12].

CP was first found in 1986, and by 1989 CP was recognized to be the third* Chlamydia* following* Chlamydia trachomatis* and* Chlamydia psittaci*. CP is distributed around the world and transmitted through aerosols in the population [10]. Three weeks after primary infection of* Chlamydia pneumoniae*, IgM antibodies can be detected. Antibodies can be detected in sera from patients with acute or past infections, and further test is required at the 2nd or 3rd week and the 6th week of onset to distinguish new infection from past infection or chronic infection. Obviously, the formation of antibody after CP infection is slower than that of other pathogens [11]. IgG antibody levels usually rise in 1-2 weeks after reinfection, but sometimes there is no reinfection. IgM antibody levels may be slightly elevated when infection reoccurs.

Roughly half of CP infections are asymptomatic or of moderate sore throat. Other CP infections are mainly manifested as sustained dry cough, headache, and fever. CP chronic infection is more common in adults. More than 50% of adults are infected with* Chlamydia pneumoniae*, producing anti-*Chlamydia* antibody. The serological analysis of 4500 healthy objects given physical examination indicated that about 50%~70% of adults had been infected. CP infection can induce specific T cell immunity and B cell immunity in human body. These infectious antibodies could temporarily provide the certain body immune protection, but the immunity is not strong enough and only maintains a short time, which in most cases fails to block the reinfection with recurrent attacks. Serological epidemiology studies have shown that only 3–5 years after infection with* Chlamydia pneumoniae*, serum antibodies will get reduced or disappear, and on the other side, population survey also has shown that the antibody level is high in crowd, which indicates that in fact almost every person is infected with CP in their life repeatedly [12]. Specific IgM and IgG antibodies of CP can be detected in healthy people, suggesting that there are healthy carriers or hidden infections, which is consistent with our results. CP is a common pathogen of infectious diseases of respiratory system in human. In recent years, CP has been found to be associated with some chronic diseases, including bronchial asthma, coronary heart disease, and atherosclerosis, as well as the relatively rare diseases, such as meningitis, myocarditis, and Guillain-Barre syndrome. Recently clinical studies have also found that CP antibodies were detected to be significantly higher than those in the corresponding control groups [4] in a variety of systemic diseases, such as pharyngitis, laryngitis, tympanitis, sinusitis, sarcoidosis, malignant lymphoma, multiple sclerosis, senile dementia, lung cancer, premature birth, and premature rupture of membranes [3, 4, 8]. CP has become a pathogen that is a serious danger to the mankind and thus increasingly draws more and more attention of scholars in the world.

## Figures and Tables

**Table 1:** 

	Spring (Feb.–Apr.)	Summer (May–Sept.)	Autumn (Oct.-Nov.)	Winter (Dec.-Jan.)
Children	375	375	375	375
Juniors	375	375	375	375
Seniors	375	375	375	375

Total	1125	1125	1125	1125

**Table 2:** Serum IgM and IgG detection results for MP and CP in groups A, B, and C number of cases (percentage %).

Group	MP positive *n* (%)			CP positive *n* (%)		
	IgM	IgG	IgM + IgG	IgM	IgG	IgM + IgG
Group A (*n* = 1500)	49 (3.25)	675 (45.00)	15 (1.00)	42 (2.80)	705 (47.00)	15 (1.00)
Group B (*n* = 1500)	30 (2.00)	630 (42.00)	12 (0.80)	57 (3.80)	750 (50.00)	23 (1.53)
Group C (*n* = 1500)	23 (1.53)	600 (40.00)	8 (0.53)	68 (4.53)	777 (51.80)	27 (1.80)
Total (*n* = 4500)	102 (2.27)	1905 (42.33)	35 (0.78)	167 (3.71)	2232 (49.60)	65 (1.44)

**Table 3:** MP and CP infections in 4500 healthy subjects under physical examination in different seasons in 2016.

Season	MP IgM positive *n* (%)	CP IgM positive *n* (%)
Spring (*n* = 1125)	28 (2.49)	45 (4.00)
Summer (*n* = 1125)	20 (1.78)	32 (2.84)
Autumn (*n* = 1125)	17 (1.51)	28 (2.49)
Winter (*n* = 1125)	37 (3.29)	60 (5.33)
